# Unveiling Novel Genetic Mutations and Prognostic Indicators in Breast Carcinoma: An Analysis of The Cancer Genome Atlas (TCGA) Data

**DOI:** 10.7759/cureus.98637

**Published:** 2025-12-07

**Authors:** Raman Butta, Shristi Butta

**Affiliations:** 1 Bioinformatics, St. Xavier's College, Mumbai, Mumbai, IND; 2 Oncopathology, Institute of Post-Graduate Medical Education and Research and Seth Sukhlal Karnani Memorial Hospital, Kolkata, IND

**Keywords:** ca breast prognostic markers, epigenetic, integrated bioinformatics analysis, oncogenesis, tcga database

## Abstract

Background

Breast carcinoma is one of the leading causes of cancer-associated mortality in women worldwide. Although several advances have been made in molecularly classifying breast cancers, treatment resistance continues to limit the overall survival. The Cancer Genome Atlas (TCGA) has unraveled diverse genomic alterations in breast carcinoma. However, some potential biomarkers still remain unexplored, like SETDB1, a histone methyltransferase involved in epigenetic silencing of tumor suppressor genes. ARMCX5 and SLCO6A1 are also some of the unexplored genes that could have a potential role in drug resistance.

Materials and methods

The Mutation Annotation Format (MAF) data set from the Cancer Genome Atlas Breast Cancer (TCGA-BRCA) cohort was analyzed using the Maftools, Survival, Mclust, and Survminer R packages. Oncodrive driver analysis and protein family (PFAM) domain mapping were performed. A total of 845 cases of breast carcinoma with complete survival data were retrieved, of which mutation data for 800 cases were available. Comprehensive mutation analysis was also done to unveil unexplored genes. Survival data of 845 cases were integrated for Kaplan-Meier and Cox proportional hazard analysis to ascertain the prognostic significance of an array of genes. Oncogenic signaling pathway mapping was done to determine the clinical enrichment of the genes associated with breast carcinoma. Genes associated with clinical enrichment, clustering of somatic mutations, and prognosis were subjected to further analysis.

Results

Besides the established molecular drivers like PIK3CA and TP53, we found several novel and understudied genes with potential prognostic and oncogenic significance. SETDB1 (p < 0.0001), USP37 (p < 0.0001), NDUFS1 (p = 0.025), TRPM4 (p < 0.0001), and MYO18A (p < 0.0001) were associated with poor prognosis. ARMCX5 (p < 0.0001) and SLCO6A1 (p < 0.0001) were enriched in high-grade tumors.

Conclusion

The TCGA-BRCA cohort analysis emphasizes a potential interplay of metabolic genes like NDUFS1, TRPM4, ARMCX5, SLCO6A1, and epigenetic axis genes like SETDB1 and USP37 in the oncogenesis and prognosis of breast carcinomas. These observations could open potential avenues for exploring novel therapeutics in aggressive breast carcinomas.

## Introduction

Breast cancer accounts to be the most common malignancy worldwide after having surpassed lung cancer [1]. It is one of the leading causes of cancer-related mortality in women worldwide. Despite early diagnosis and major advances in targeted therapeutics, it continues to have a dismal prognosis, due to tumor heterogeneity and therapeutic resistance. The genetic and epigenetic traits of the tumor cells may contribute to their proliferative potential, tumor invasiveness, and therapy resistance [2]. Current therapy regimens are based on the molecular classification of breast carcinomas and essentially depend on the hormone receptor (estrogen receptor (ER), progesterone receptor (PR), and human epidermal growth factor receptor 2 (HER2)) status and proliferative indices (Ki67) of the breast carcinoma [3]. However, the molecular diversity and intratumoral complexity of breast cancer often lead to therapeutic failure. Hence, recognizing novel and under-reported genomic signatures could open newer avenues to effective targeted therapeutics.

The Cancer Genome Atlas Breast Cancer (TCGA-BRCA) dataset provides a detailed and extensive genomic landscape of breast carcinomas [4]. The TCGA-BRCA identifies oncogenes and tumor suppressor genes implicated in tumorigenesis and oncogenic pathways like the PI3K/AKT/mTOR pathway, the Tp53-mediated tumor suppressor pathway, and the cell adhesion regulation pathway. Despite a range of somatic mutations identified in TCGA, there remain a few under-reported genes that could hold plausible prognostic and therapeutic relevance.

Mitochondrial-metabolic pathways of late have emerged as adaptive mechanisms in reprogramming tumor cells to a hypoxic tumor microenvironment [5]. Further, epigenetic modification through histone methyltransferases and deubiquitinases is also implicated in oncogenesis [6]. Furthermore, altered ion channel activity and cell adhesion-cytoskeleton modification are also associated with tumor invasiveness and metastasis.

Herein, we utilize the large-scale genomic data from the TCGA-BRCA dataset for comprehensive mutational profiling of invasive breast carcinomas. Besides the known driver genes, some novel under-reported genes have also been analyzed. Many of these less-reported genes are involved in the mitochondrial metabolic pathways, ion signaling pathways, and epigenetic regulation. This study utilizes the Mutation Annotation Format (MAF) data from the TCGA-BRCA cohort to perform an integrative mutation analysis. The study aims to ascertain potential under-reported molecular players involved in the oncogenesis and prognosis of breast carcinomas.

## Materials and methods

This was a retrospective study conducted on the publicly available dataset TCGA-BRCA. The MAF data set from the TCGA-BRCA cohort was downloaded from the Genomic Data Commons (GDC) Data Portal (https://portal.gdc.cancer.gov/). Clinical data were extracted from the Clinica.tsv file, for analyzing parameters like demographics, pathology, and survival. This data was matched sample to sample with the TCGA-BRCA cohort.

A total of 845 cases of breast carcinoma with complete survival data were retrieved from the TCGA-BRCA cohort, of which mutation data were available for 800 cases. Cases with missing clinical data were excluded from the survival analysis. Thus, survival analysis was conducted on 845 cases, whereas mutation analysis was restricted to 800 cases.

Preprocessing

The compressed MAF file was analyzed using the R version 4.3.1 (R Foundation for Statistical Computing, Vienna, Austria) and processed using the maftools package (version 2.16.0). Maftools is a contemporary cancer genomic package used for large-scale datasets [7].

Analytical workflow included structured mutation profiling, variant classification, tumor heterogeneity assessment, and survival endpoint analysis, emphasizing on integration of genomic alterations with clinical and survival outcomes. These could potentially open newer avenues for future multi-omics extension and experimental validation.

Mutation profiling

Comprehensive mutation analysis was also done to unveil unexplored genes besides the known oncodrivers. Oncoplots were generated for the top 10 mutated genes across samples using oncoplot(). Transition-Transversion ratio was found using the titv() function, and visualization was done with plotTiTv(), to assess the genetic mutation signatures. Lollipop plots were generated for oncogenes using lollipopPlot() to identify amino acid-level mutations, thereby highlighting hotspot domains. Rainfall plots were generated using the rainfallPlot() function to find out localized hypermutated events (kataegis). Potential oncodrivers were assessed using the oncodrive() function.

Genes that had clustered mutation patterns and a false discovery rate (FDR) < 0.1 were considered statistically significant oncodrivers. The protein family (PFAM) database via the pfamDomains() function was used to assess recurrently affected domains of the proteins. Heatmaps were generated using the somaticInteractions() function to depict co-occurrence and mutual exclusivity (p-value thresholds: 0.05 and 0.1). Pathway-level enrichment was performed using the pathways() and plotPathways() functions, and mutation load was compared with other TCGA tumors using the tcgaCompare() function.

Tumor heterogeneity assessment

Tumor heterogeneity was assessed using the inferHeterogeneity() function. MATH (Mutant-Allele Tumor Heterogeneity) score was computed. Higher MATH scores (>50) were considered to indicate increased tumor clonal diversity and potential therapeutic resistance.

Survival analysis

Outcomes were assessed using the survival (v3.6) and survminer (v0.4.9) functions. Overall survival (OS) was considered time of diagnosis till death or the last follow-up of the patient. The Kaplan-Meier method was used to assess the survival using the survfit() function, and the survival difference between mutant and wild-type groups was analyzed using the log-rank test (p < 0.05 considered statistically significant). Univariate Cox proportional hazards models were used to calculate hazard ratios (HRs).

Statistical analysis

Statistical analyses were performed using R version 4.3.1. A p-value < 0.05 was considered statistically significant.

## Results

**Figure 1:**
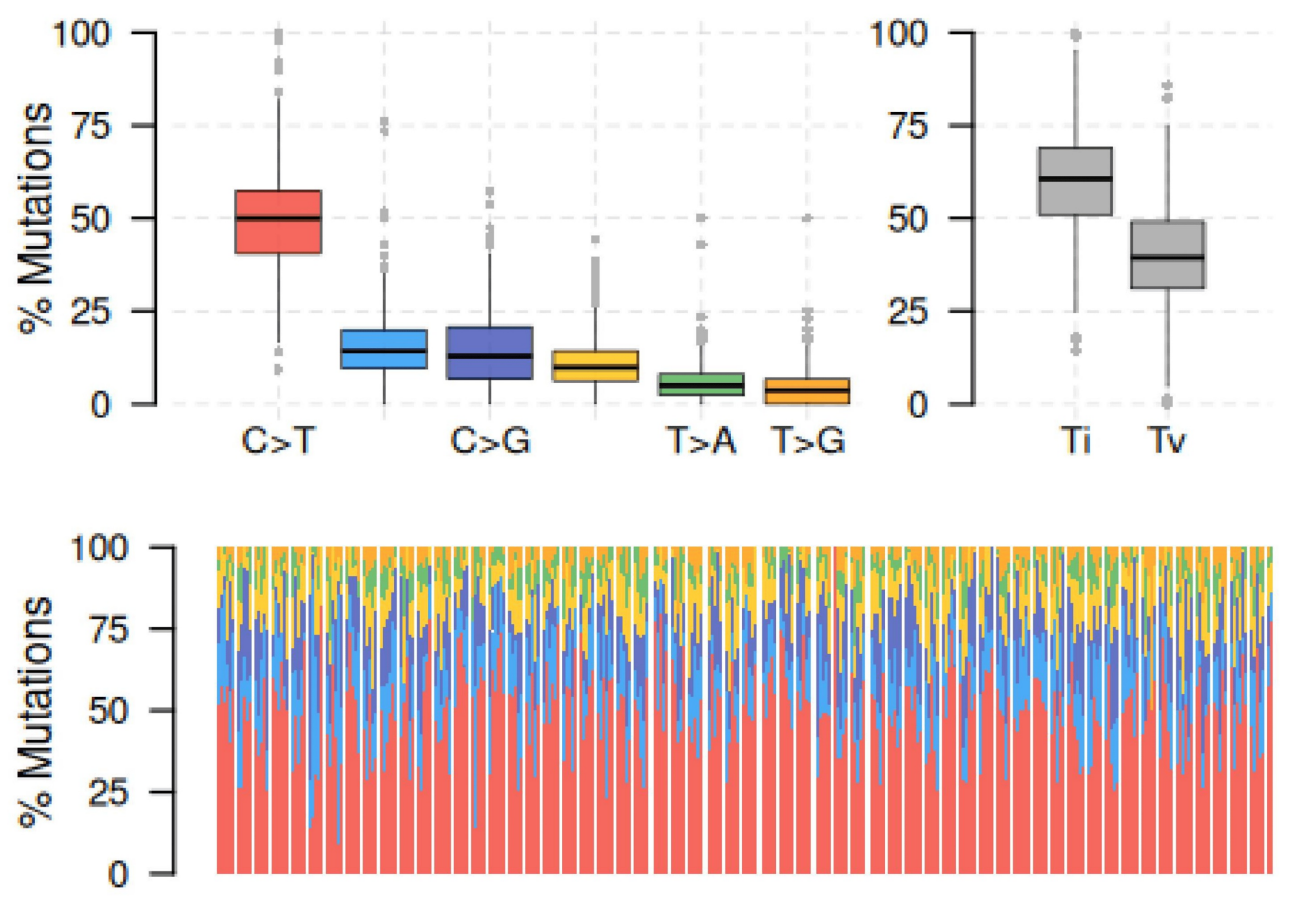
Transition-Transversion Plot Mutations classified as single-nucleotide polymorphisms (SNPs) are categorized into six classes based on the type of nucleotide substitution: transitions (Ti): purine-to-purine (A↔G) or pyrimidine-to-pyrimidine (C↔T), and transversions (Tv): purine-to-pyrimidine or vice versa (A↔C, A↔T, G↔C, G↔T). The majority were SNPs, primarily involving a C > T transition, which was consistent with aging-related mutational burden.

A total of 845 cases of breast carcinoma with complete survival data were retrieved from the TCGA-BRCA cohort, of which mutation data were available for 800 cases. A median of 34 variant mutation burden per case was noted. The most common mutation type was a missense mutation, which was followed by nonsense and frame-shift deletions. The majority were single-nucleotide polymorphisms (SNPs), primarily involving a C > T transition, which was consistent with aging-related mutational burden (Figure 1). The most commonly mutated genes in the cohort were PIK3CA (35%), TP53 (34%), TTN (17%), CDH1 (14%), GATA3 (13%), MAP3K1 (10%), KMT2C (9%), MUC16 (9%), HMCN1 (6%), and FLG (6%). These genetic mutations affected 83.7% of the tumors analysed. The average transition-transversion ratio (Ti/Tv) was 2.3.

Identification of driver genes

**Figure 2:**
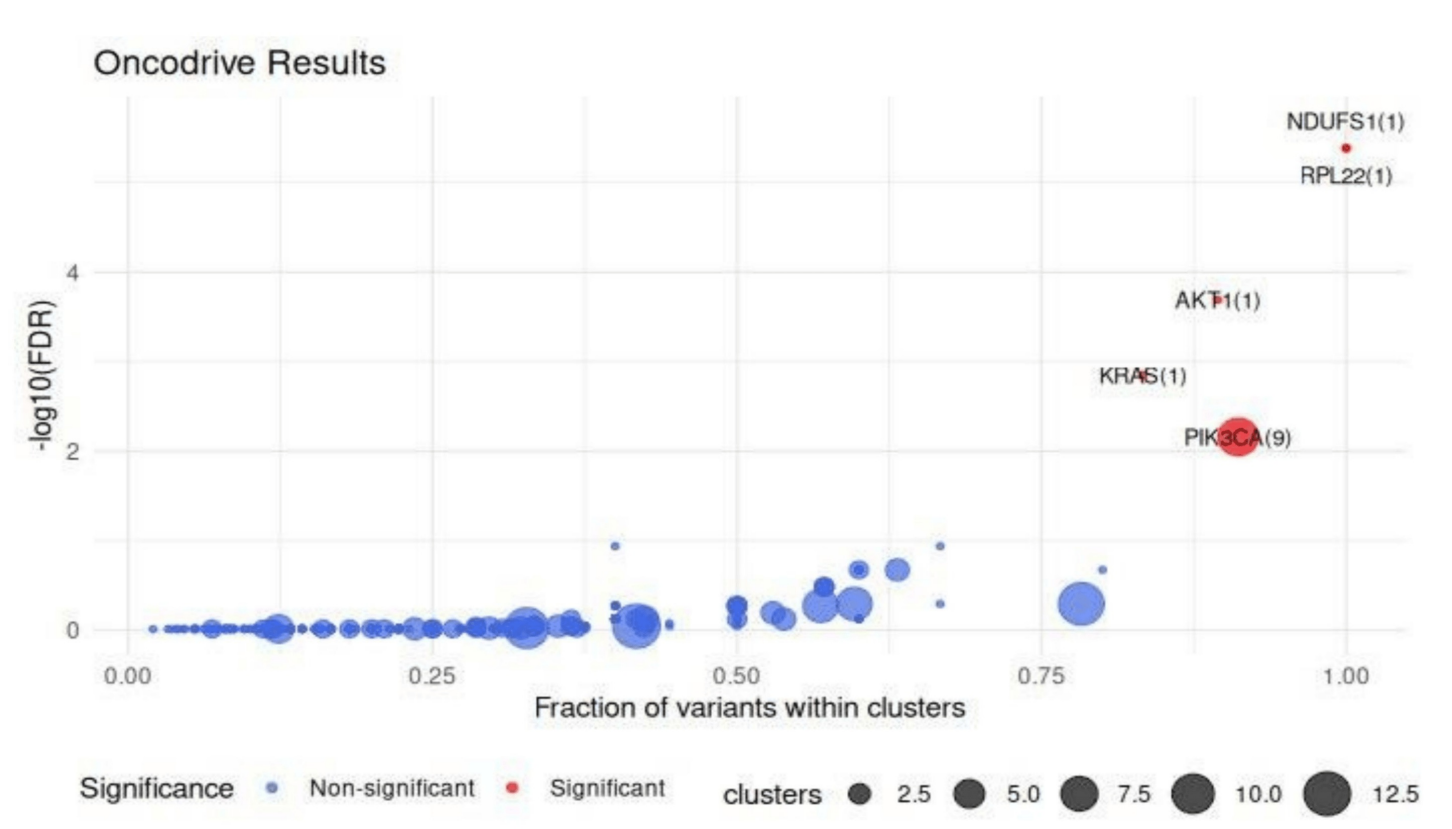
Oncodriver Plot A false discovery rate (FDR) cutoff of 0.1 is set to highlight genes with statistically significant clustering of mutations. NDUFS1 and RPL22 are significant driver genes (FDR < 0.1).

Oncodrive algorithm found a number of significantly clustered genes (FDR < 0.1), which was consistent with oncodriver-like mutational behavior. PIK3CA and TP53 showed the strongest clustering patterns. However, the analysis also identified some under-reported genes like NDUFS1 and RPL22 as statistically significant potential genes (FDR < 0.1) (Figure 2).

To ascertain the clinical importance, mutation data were integrated with survival (n = 845). Kaplan-Meier and Cox proportional hazards analyses disclosed a number of under-reported genes with statistically significant prognostic outcomes.

**Figure 3:**
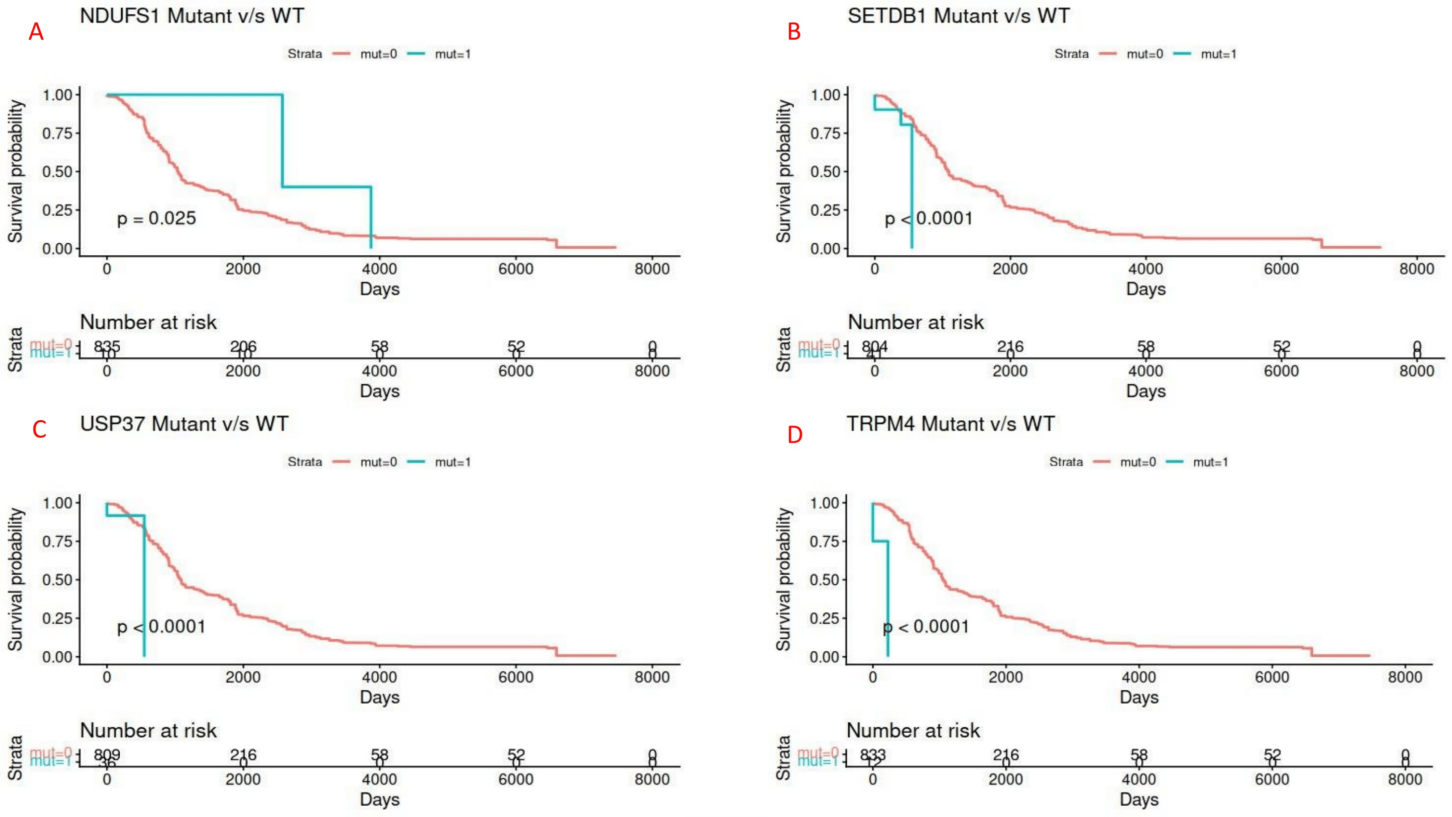
Survival Analysis of Under-Reported Genes NDUFS1 gene alterations were associated with statistically significant poor overall survival (p = 0.025). SETDB1 was associated with poor prognosis (p < 0.0001). USP37 revealed a poor survival outcome (p < 0.0001). TRPM4 was significantly associated with reduced survival outcome (p < 0.0001).

NDUFS1 gene alterations were associated with statistically significant poor OS (p = 0.025). NDUFS1 gene alterations revealed low median survival compared to its wild-type equivalents. A histone methyltransferase, SETDB1, was associated with poor prognosis (p < 0.0001). Recent data suggest that SETDB1 alterations are associated with epigenetic reprogramming and can hence serve as a potential therapeutic target. A deubiquitinase controlling cyclin A stability, USP37, revealed poor survival outcome (p < 0.0001). A calcium-activated cation channel, TRPM4, was significantly associated with reduced survival outcome (p < 0.0001) (Figure 3). TRPM4 enrichment in high-grade tumors highlights the role of ion channels in oncogenesis. MYO18A, encoding a myosin motor protein, also revealed poor survival association (p < 0.0001).

**Figure 4:**
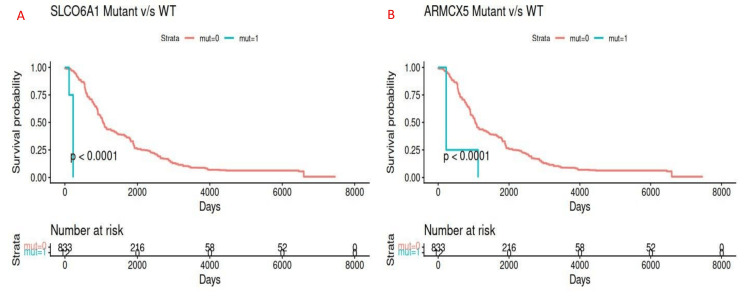
Survival Analysis of SLCO6A1 and ARMCX5 Genes ARMCX5 and SLCO6A1 genes revealed statistically significant reduced survival outcomes (p < 0.0001).

ARMCX5 and SLCO6A1 genes revealed statistically significant clinical enrichment in high-grade (grade III-IV) tumors (FDR < 0.01). Additionally, these were associated with reduced survival outcomes (p < 0.0001) (Figure 4). ARMCX5, a mitochondrial trafficking protein, was mutated in about 4.1% of grade IV tumours and 0.7% of grade II-III tumors. SLCO6A1, a member of the solute carrier family protein, was found to be mutated in 3.6% of grade IV and 0.5% of lower-grade tumors. SLCO6A1 and TRMP4 alterations in high-grade tumors highlight the potential role of cancer transportome in chemotherapeutic resistance and tumor progression.

Analysis of tumor heterogeneity using inferHeterogeneity() revealed MATH scores > 50 in patients possessing NDUFS1 and SETDB1 mutations, thus showing high intratumoral heterogeneity (p = 0.01).

Recent molecular advancements in breast carcinoma pathogenesis have highlighted the role of epigenetic modifiers, mitochondrial regulators, and membrane transporters. Consistent with these advancements, our analysis reveals SETDB1, NDUFS1, ARMCX5, TRPM4, and SLCO6A1 as significantly altered genes in high-grade breast carcinomas.

## Discussion

This study provides a comprehensive analysis of the diverse mutation profile of invasive breast carcinoma from the TCGA-BRCA cohort, thus unravelling unexplored genomic signatures. Maftools bioinformatics framework has been used to integrate and analyze driver mutations and prognostic determinants. In addition to the known oncodrivers like PIK3CA and TP53, under-reported mutations were also identified. Some of the infrequently reported genes included NDUFS1, SETDB1, USP37, TRPM4, MYO18A, ARMCX5, and SLCO6A1. These demonstrated statistically significant prognostic and biologic relevance. Hence, this study reveals significant metabolic and epigenetic interplay in breast carcinoma pathogenesis. Additionally, it also helps in understanding the mutational landscape of breast carcinomas, in turn identifying the cause behind the increasing therapeutic resistance.

Previous large-scale studies like METABRIC and TCGA-BRCA have highlighted the mutational spectra of the invasive breast carcinomas and thus have played a key role in classifying the molecular subtypes of breast carcinomas [8]. However, some genes remain under-reported, and identifying these unexplored mutations could potentially open newer avenues to targeted therapeutics in resistant breast carcinomas.

Amongst the unexplored genes, NDUFS1 has unfolded as a potential metabolic driver gene with poor survival outcome. The NDUFS1 gene encodes a subunit of NADH:ubiquinone oxidoreductase or the mitochondrial complex I. This is implicated in oxidative phosphorylation. A mutation in NDUFS1 can, in turn, lead to impairment of electron transport and hence generation of excessive reactive oxygen species, resulting in tumor progression [9]. Vyas et al. found that defects in mitochondrial complex I promote a shift to the glycolytic pathway or Warburg effect and result in tumor aggressiveness [10]. The enrichment of mutations in NDUSF1 and ARMCX5 in high-grade tumors potentially indicates their role in the metastasis of breast carcinomas.

Further, epigenetic modifiers like SETDB1 and USP37 have also shown prognostic significance. SETDB1 encodes a histone methyltransferase that is implicated in H3K9 trimethylation. This is associated with heterochromatin formation and transcriptional repression. SETDB1 has been associated with epigenetic silencing of tumor suppressor genes and has also been implicated in stem cell-like properties, particularly in melanoma, lung carcinomas, and invasive breast carcinomas [11]. Additionally, SETDB1 overexpression has been associated with Luminal B and triple-negative molecular subtypes of breast carcinoma. Both of these are associated with an aggressive clinical course and a poor prognosis, thus implicating their role in therapy resistance [12]. In addition, SETDB1 impairs DNA damage repair and hence increases mutation burden and results in chromosomal instability [13]. This genomic instability could be a plausible explanation for poor survival and tumor heterogeneity as analyzed in this study.

Furthermore, mutations in TRPM4 and MYO18A were also observed to have onco-driver properties. TRPM4 encodes a calcium-activated cation channel. Its overexpression has been implicated in several solid malignancies like breast and prostate carcinomas. It has been associated with epithelial-mesenchymal transition and metastasis. Recent studies have also highlighted that TRPM4 is implicated in chemoresistance in breast carcinomas [14]. Mutations in TRPM4 likely disturb the ion homeostasis, thus influencing the calcium-dependent signalling cascade. This, in turn, leads to increased sodium influx and membrane depolarisation, leading to sustained oncogenesis. MYO18A is a member of the myosin family involved in actin remodelling and cytoskeletal organization. MYO18A mutations are implicated in increasing cell motility and metastasis [15]. Hence, metastatic dissemination associated with MYO18A mutations supports the argument of reduced survival outcomes, as seen in this study.

Additionally, this study highlights two understudied genes in breast carcinoma - ARMCX5 and SLCO6A1. The ARMCX5 gene belongs to the ARMCX family and is located on the X chromosome. These are localized on the outer mitochondrial membrane and are implicated in mitochondrial trafficking and cell survival regulation. It stabilizes mitochondrial membrane potential and protects against apoptosis, which in turn confers a survival advantage under hypoxic conditions [16]. In addition, SLCO6A1 is a member of the solute carrier organic anion transporter family. It is mediated in sodium-independent uptake of large anionic molecules across the plasma membrane [17]. This study has shown significant clinical enrichment of ARMCX5 and SLCO6A1 in high-grade tumors. Additionally, both of these have shown poor survival outcomes. This pattern suggests that both ARMCX5 and SLCO6A1 have a plausible role in tumor aggressiveness and therapeutic resistance.

However, this study has limitations owing to its retrospective design and usage of publicly available data from the TCGA-BRCA cohort study. Further, this study is solely based on the bioinformatics analysis of TCGA-BRCA datasets without experimental validation. Further studies are essential for immunohistochemical or proteomic level validation of the genetic mutations derived from the study. The TCGA-BRCA cohort represents a heterogeneous study population with variable molecular subtypes and ethnicity. This may thus confound the frequency of mutations and survival outcomes across molecular subtypes and ethnic backgrounds. Additionally, multi-omics validation is essential to establish the functional significance of the derived mutational alterations for prognostic impact and targeted therapeutics.

## Conclusions

This study provides insights into unexplored and understudied genes in breast carcinoma with potential oncogenic and prognostic impact. It suggests that interplay between metabolic, genomic, and epigenetic pathways forms a plausible basis for tumor evolution and therapy resistance. The results derived from the bioinformatic analysis require further experimental studies to confirm functional relevance.

## References

[1] Sung H, Ferlay J, Siegel RL, Laversanne M, Soerjomataram I, Jemal A, Bray F (2021). Global cancer statistics 2020: GLOBOCAN estimates of incidence and mortality worldwide for 36 cancers in 185 countries. CA Cancer J Clin.

[2] Lüönd F, Tiede S, Christofori G (2021). Breast cancer as an example of tumour heterogeneity and tumour cell plasticity during malignant progression. Br J Cancer.

[3] Carvalho E, Canberk S, Schmitt F, Vale N (2025). Molecular subtypes and mechanisms of breast cancer: precision medicine approaches for targeted therapies. Cancers (Basel).

[4] Cancer Genome Atlas Network (2012). Comprehensive molecular portraits of human breast tumours. Nature.

[5] Martínez-Reyes I, Chandel NS (2020). Mitochondrial TCA cycle metabolites control physiology and disease. Nat Commun.

[6] Dawson MA, Kouzarides T (2012). Cancer epigenetics: from mechanism to therapy. Cell.

[7] Mayakonda A, Lin DC, Assenov Y, Plass C, Koeffler HP (2018). Maftools: efficient and comprehensive analysis of somatic variants in cancer. Genome Res.

[8] Curtis C, Shah SP, Chin SF (2012). The genomic and transcriptomic architecture of 2,000 breast tumours reveals novel subgroups. Nature.

[9] Hoefs SJ, Skjeldal OH, Rodenburg RJ (2010). Novel mutations in the NDUFS1 gene cause low residual activities in human complex I deficiencies. Mol Genet Metab.

[10] Vyas S, Zaganjor E, Haigis MC (2016). Mitochondria and cancer. Cell.

[11] Sun QY, Ding LW, Xiao JF (2015). SETDB1 accelerates tumourigenesis by regulating the WNT signalling pathway. J Pathol.

[12] Liu Z, Liu J, Ebrahimi B (2022). SETDB1 interactions with PELP1 contributes to breast cancer endocrine therapy resistance. Breast Cancer Res.

[13] Micallef I, Fenech K, Baron B (2024). Therapeutic targeting potential of the protein lysine and arginine methyltransferases to reverse cancer chemoresistance. Front Mol Biosci.

[14] Guo J, Liu F, Yang X (2025). TRPM4 channels contribute to Adriamycin chemoresistance in breast cancer cells. Eur J Pharmacol.

[15] Buschman MD, Field SJ (2018). MYO18A: an unusual myosin. Adv Biol Regul.

[16] Gan Y, Zhong F, Wang H, Li L (2022). The valuable role of ARMC1 in invasive breast cancer as a novel biomarker. Biomed Res Int.

[17] Telfer-Sutherland R, Page L, Meeson A, Lowes S (2025). Differential expression patterns of SLCO solute carriers in human breast cancer cell lines and tumour samples. J Cell Mol Med.

